# Integrated characterization of SARS-CoV-2 genome, microbiome, antibiotic resistance and host response from single throat swabs

**DOI:** 10.1038/s41421-021-00248-3

**Published:** 2021-03-30

**Authors:** Bo Lu, Yi Yan, Liting Dong, Lingling Han, Yawei Liu, Junping Yu, Jianjun Chen, Danyang Yi, Meiling Zhang, Xin Deng, Chao Wang, Runkun Wang, Dengpeng Wang, Hongping Wei, Di Liu, Chengqi Yi

**Affiliations:** 1 State Key Laboratory of Protein and Plant Gene Research, School of Life Sciences, Peking University, Beijing, 100871 China; 2 Peking-Tsinghua Center for Life Sciences, Peking University, Beijing, 100871 China; 3 CAS Key Laboratory of Special Pathogens, Wuhan Institute of Virology, Center for Biosafety Mega-Science, Chinese Academy of Sciences, Wuhan, Hubei 430071 China; 4 National Virus Resource Center, Wuhan Institute of Virology, Chinese Academy of Sciences, Wuhan, Hubei 430071 China; 5 Computational Virology Group, Center for Bacteria and Viruses Resources and Bioinformation, Wuhan Institute of Virology, Chinese Academy of Sciences, Wuhan, Hubei 430071 China; 6 University of Chinese Academy of Sciences, Beijing, 101409 China; 7 GrandOmics Biosciences, Wuhan, Hubei 430000 China; 8 Department of Gastroenterology, The First Medical Center of PLA General Hospital/Chinese PLA Postgraduate Military Medical School, Beijing, 100853 China; 9 Department of Biomedical Sciences, City University of Hong Kong, Kowloon Tong, Hong Kong, China; 10 First Affiliated Hospital of Xinjiang Medical University, Urumqi, Xinjiang 830054 China; 11 Department of Chemical Biology and Synthetic and Functional Biomolecules Center, College of Chemistry and Molecular Engineering, Peking University, Beijing, 100871 China

**Keywords:** Transcriptomics, Transposition

## Abstract

The ongoing coronavirus disease 2019 (COVID-19) pandemic, caused by severe acute respiratory syndrome coronavirus 2 (SARS-CoV-2) infection, poses a severe threat to humanity. Rapid and comprehensive analysis of both pathogen and host sequencing data is critical to track infection and inform therapies. In this study, we performed unbiased metatranscriptomic analysis of clinical samples from COVID-19 patients using a recently developed RNA-seq library construction method (TRACE-seq), which utilizes tagmentation activity of Tn5 on RNA/DNA hybrids. This approach avoids the laborious and time-consuming steps in traditional RNA-seq procedure, and hence is fast, sensitive, and convenient. We demonstrated that TRACE-seq allowed integrated characterization of full genome information of SARS-CoV-2, putative pathogens causing coinfection, antibiotic resistance, and host response from single throat swabs. We believe that the integrated information will deepen our understanding of pathogenesis and improve diagnostic accuracy for infectious diseases.

## Introduction

Longstanding, emerging, and re-emerging infectious diseases continuously threaten human health across centuries^1^. Precise and rapid identification of pathogens from clinical samples is important for both guiding infection treatment strategies and monitoring novel infectious disease outbreaks, e.g., the outbreak of SARS-CoV-2, in the community. While most nucleic acid amplification-based and pathogen-specific antibody detection-based molecular techniques only detect a limited number of pathogens and need their prior knowledge, metagenomic or metatranscriptomic approaches allow for comprehensive and unbiased identification and characterization of microbiome directly from clinical specimens^2^.

Compared to metagenomic sequencing, metatranscriptomic sequencing has several distinct advantages: it permits detection of RNA viruses that would not be interpreted in metagenomic data, reveals transcriptionally active organism(s) which are more etiologically important, and indicates host immune response which is essential to distinguish true pathogens from colonizers^3–5^. However, the laborious and time-consuming steps in traditional RNA sequencing (RNA-seq) experiments hinder the development of metatranscriptomics-based clinical diagnostics for rapid pathogen identification.

Very recently, we and others have independently developed a rapid and cost-effective RNA-seq method, based on Tn5 tagmentation activity towards RNA/DNA hybrids^6,7^. Our method, termed “TRACE-seq”, enables rapid one-tube library construction for RNA-seq experiments and shows excellent performance in comparison to traditional RNA-seq methods. We thus envisioned that this convenient and sensitive method could be applied to clinical specimens for unbiased metatranscriptomic analysis. In this study, we modified the TRACE-seq procedure, shortened the total time, and optimized analytical pipeline to meet the needs for clinical metatranscriptomic diagnosis and analysis. We then applied TRACE-seq to metatranscriptomic sequencing of single throat swab specimens from COVID-19 patients and healthy individuals. We found library construction of specimens could be accomplished in ~2 h with high quality. Analysis of TRACE-seq metatranscriptomic data of 13 SARS-CoV-2-positive samples and 2 negative samples demonstrated the success of this method to sensitively detect SARS-CoV-2 with high coverage even for samples with low virus abundance, or to assemble unknown microbe genome de novo (using SARS-CoV-2 as an example). Moreover, TRACE-seq sensitively detected the microbiome and simultaneously allowed for interrogating antibiotic resistance and host responses. Taken together, TRACE-seq enables unbiased pathogen detection and could have broad applications in the metatranscriptomic study and clinical diagnosis.

## Results

### TRACE-seq enables metatranscriptomic analysis

**Fig. 1:**
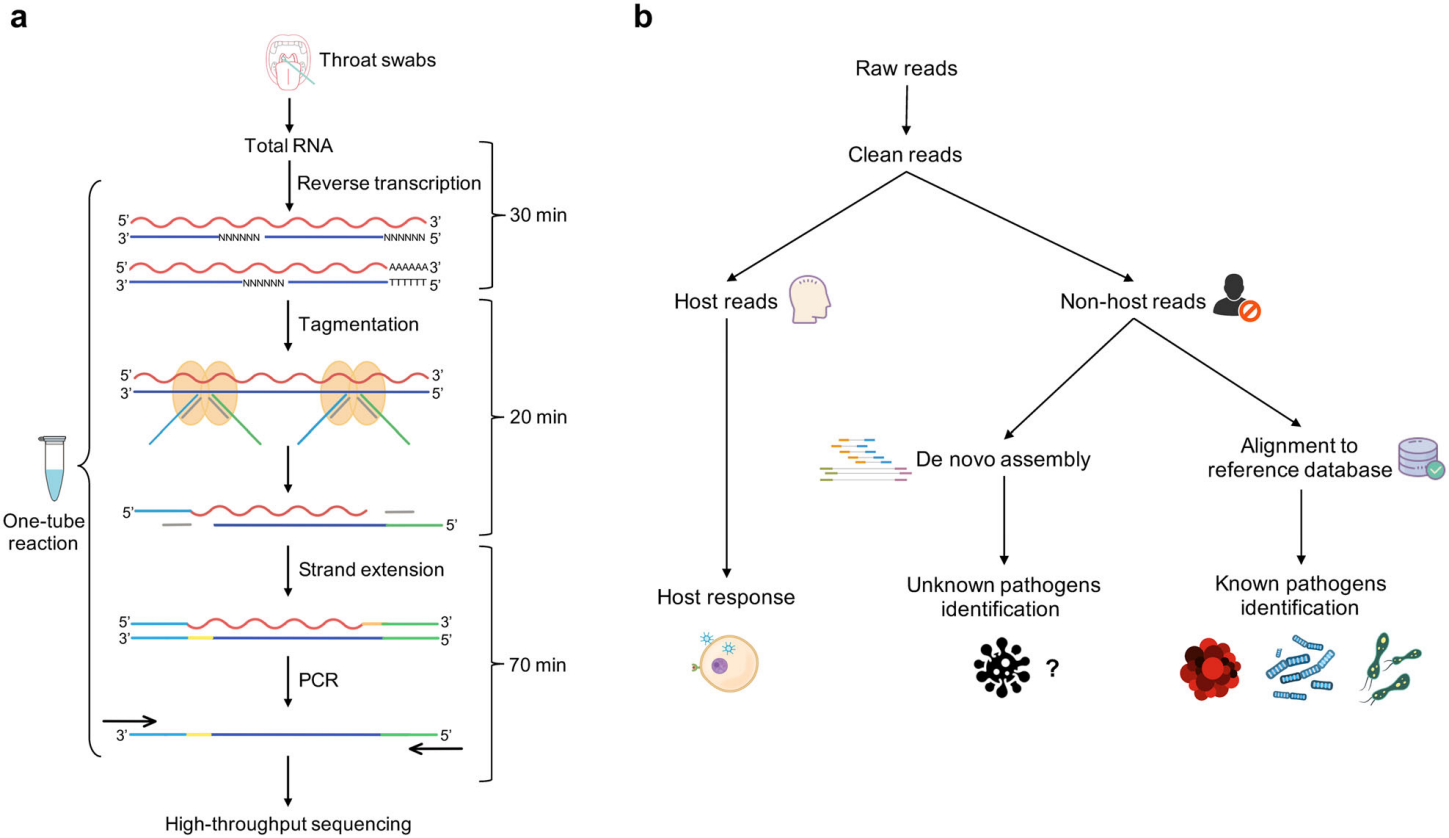
Workflow of TRACE-seq enables metatranscriptomic sequencing for clinical diagnosis. **a** A wet lab protocol of TRACE-seq starting with total RNA extracted from throat swabs of COVID-19 patients. **b** A dry lab pipeline including known and unknown pathogen identification and host response characterization.

To perform metatranscriptomic analysis on clinical samples, such as throat swabs in this study, we made several modifications to TRACE-seq. First, to achieve unbiased sequencing of microbiome, we used both random hexamer and oligo d(T)_23_VN primers for reverse transcription, using approximately 1/10 total RNA extracted from a single throat swab as input. Secondly, we reduced the total time of library construction to around 2 h (Fig. 1a), which enables TRACE-seq to be more compatible for clinical use, especially when substantial numbers of specimens require investigation. Third, we developed a tailored analytical pipeline of TRACE-seq to simultaneously identify known and unknown pathogens and at the meanwhile to characterize host transcriptional response in a single metatranscriptomic profiling reaction (Fig. 1b). This new pipeline allowed us to obtain rich information from the metatranscriptomic data generated by the modified TRACE-seq.

### Sensitive detection of SARS-CoV-2 genome

**Fig. 2:**
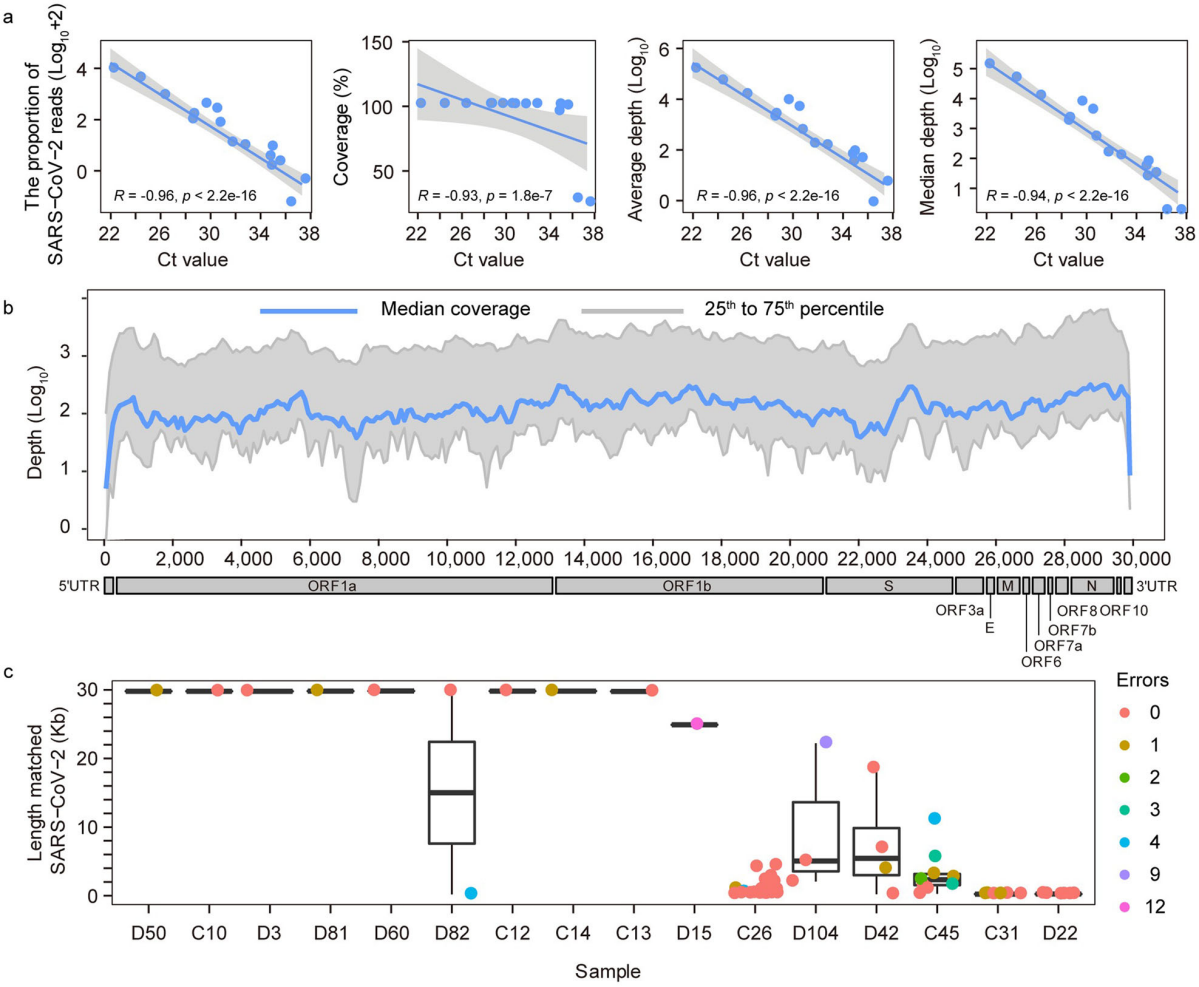
Genome coverage of SARS-CoV-2. **a** Correlation between SARS-CoV-2 sequencing data and Ct values. From the left to the right: the correlation between the ratio of SARS-CoV-2 reads, the coverage of SARS-CoV-2 genome, the average sequencing depth, the median sequencing depth, and the Ct value of each sample. Linear regression indicates the relationship between the sequencing data and the Ct value of samples. **b** Genome coverage of sequenced samples across the SARS-CoV-2 genome. The *x*-axis represents the viral genome position, the *y*-axis represents the log_10_ depth of each site. Lines in blue represent the median sequencing depth, and areas in gray represent 25th to 75th percentile of sequencing depth. **c** De novo assembly results of SARS-CoV-2. The *x-*axis represents each sample, and the *y*-axis represents log_10_ lengths of contigs matching SARS-CoV-2. Boxplots represent the length distribution of contigs matching SARS-CoV-2. Dots in different colors represent the number of error bases (shown in legends) in each contig relative to previously known genome sequences.

Since the throat swab samples were from patients with confirmed or suspected COVID-19, we asked whether the untargeted metatranscriptomic sequencing could yield a full genome sequence of SARS-CoV-2 virus. After removing low-quality reads and human reads, the remaining reads were mapped to the SARS-CoV-2 reference genome Wuhan-Hu-1 (accession number: NC_045512). Sequencing covered the reference genome from 7134 bp to 29,903 bp (23.86%–100%), with a genome-wide average sequencing depth from 0.69× to 129,901× (Supplementary Table S1). Subsequent correlation analysis revealed that the proportion of obtained reads of SARS-CoV-2, the coverage to the reference genome, the average sequencing depth and the median sequencing depth all showed a significant negative correlation with the cycle threshold (Ct) value of SARS-CoV-2 in all samples (Spearman test, *P* < 0.01) (Fig. 2a). In addition, the nearly whole genome sequence (> 99%) could be acquired from mapping-based approach for most samples with the Ct value below 35 (*n* = 12, 85.7% of 14 samples with the Ct value below 35), with the average sequencing depth of 16,900× (from 28.3× to 129,901×, median depth 1894.96×). Moreover, in 7 samples (50%), genomes were completely covered (Fig. 2b; Supplementary Fig. S1 and Table S1).

### Reconstruction of full-length genome of SARS-CoV-2

To determine the accuracy of this method in de novo acquisition of pathogen genome, after de novo assembly from non-human reads and BLAST, contigs matching SARS-CoV-2 with a length ≤ 100 bp were discarded, and thus 64 contigs were determined to be SARS-CoV-2 genomes or genome fragments. These contigs ranged from 185 bp to 29,835 bp in length, with an average length of 6437 bp. As the Ct value of SARS-CoV-2 increases, SARS-CoV-2 contigs tended to be much more in number and shorter in length. Most of contigs (46, 71.9%) were exactly the same with our previously known viral genomes (Fig. 2c), and some (10, 15.6%) had 1 base difference (mismatch or gap) and the rest had 2–12 base differences. In samples with SARS-CoV-2 Ct value lower than 32, almost full-length genome (29,776–29,835 bp) were obtained just from de novo assembly. Thus, TRACE-seq could enable the de novo assembly of the complete genome of unknown pathogens and be readily utilized to identify emerging pathogens in patients with unknown etiology of infection and efficiently complement routine diagnostics.

### Unbiased identification of putative pathogens in addition to SARS-CoV-2

**Fig. 3:**
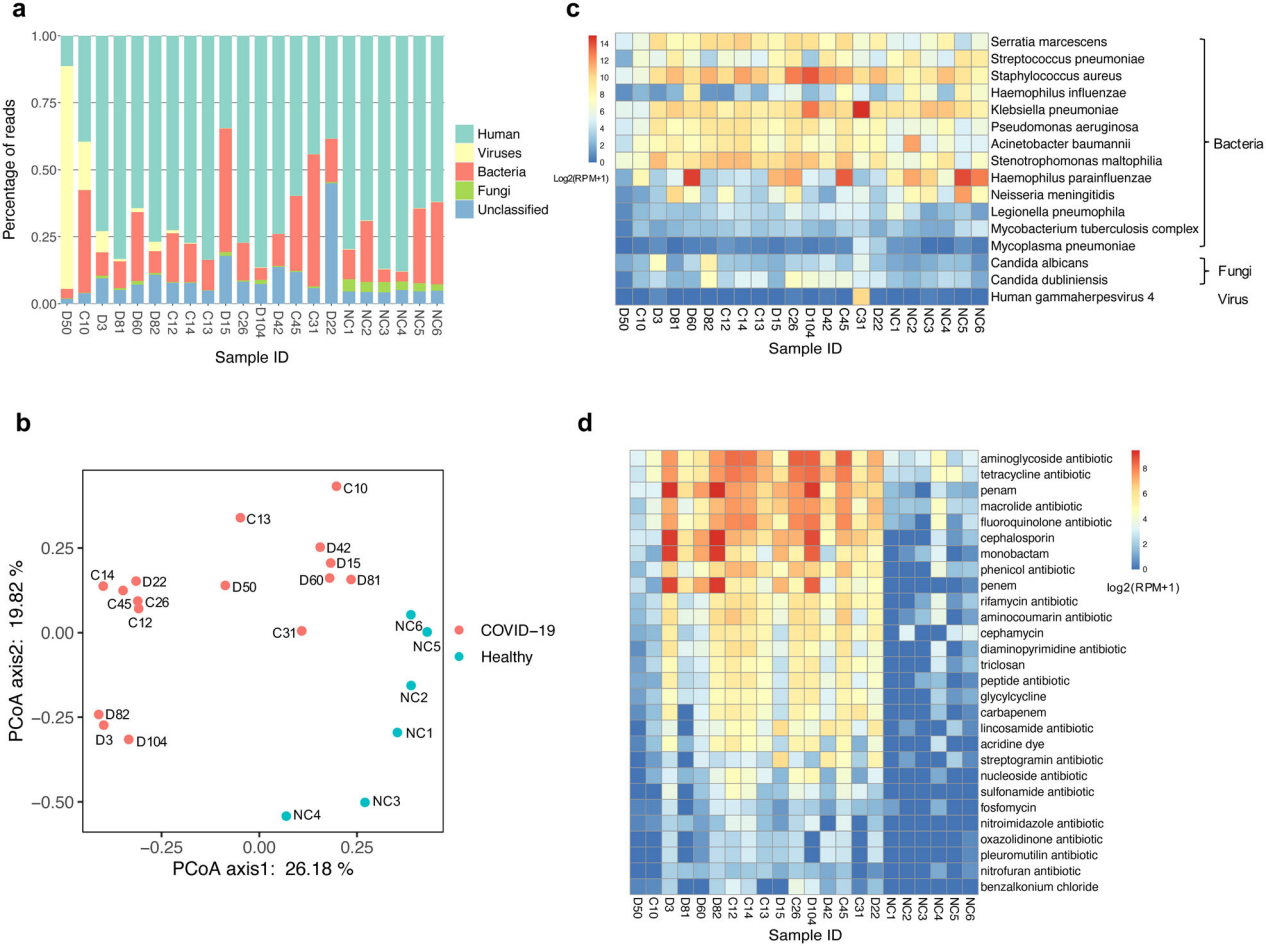
Microbiome profiles in COVID-19 patients and healthy individuals. **a** Histogram showing percentage of reads mapping to human, viruses, bacteria, and fungi for the individual samples. **b** PCoA of microbiome using relative abundance at the genus level. **c** Heatmap showing relative abundance of potential respiratory pathogens identified in SARS-CoV-2-positive and -negative samples. RPM, reads per million non-host reads. **d** Heatmap displaying relative abundance of antibiotic resistance genes in SARS-CoV-2-positive and -negative samples.

It is widely reported that coinfection (multi-species infection) contributes to enhanced morbidity and mortality, especially in elderly and immunosuppressed influenza patients^8,9^. Thus, we were curious to see if our metatranscriptomic sequencing approach could capture other pathogens in addition to SARS-CoV-2. Indeed, alignment of TRACE-seq data to microbe reference databases identified many bacteria, fungi, and viruses in both patient and healthy samples (Fig. 3a). To assess whether COVID-19 patients and healthy individuals have different microbe community in their throat, principal coordinates analysis (PCoA) was conducted using relative abundance of the microbiome. We observed that COVID-19 patients harbored a throat microbiome quite different from that of healthy individuals (Fig. 3b). The relative abundance of probable respiratory pathogens was further investigated.

Among the probable respiratory pathogens listed in Fig. 3c, *Stenotrophomonas maltophilia*, *Haemophilus parainfluenzae*, *Staphylococcus aureus*, *Streptococcus pneumoniae*, *Haemophilus influenzae*, and *Acinetobacter baumannii* are common commensal organisms of the normal oropharynx; however, they can also become opportunistic pathogens and cause infectious diseases, such as endocarditis, bacteremia, and pneumonia^10–13^. *Serratia marcescens*, *Klebsiella pneumoniae*, *Stenotrophomonas maltophilia*, *Pseudomonas aeruginosa*, *Neisseria meningitidis*, and *Legionella pneumophila* cause disease infrequently in normal hosts but can be a major cause of infection in patients with underlying or immunocompromising conditions^14–19^. *Mycoplasma pneumoniae* is a type of “atypical” bacteria that commonly causes mild infections of the respiratory system^20^. The *Mycobacterium tuberculosis* complex (MTC or MTBC) is a genetically related group of *Mycobacterium* species that can cause tuberculosis in humans or other animals^21^. As for identified fungi, *Candida dubliniensis* and *Candida albicans* are both opportunistic yeast and can be detected in the gastrointestinal tract in healthy adults; they were also known to cause respiratory diseases^22–24^. Human gammaherpesvirus 4 is one of the most common viruses in human. It is best known as the cause of infectious mononucleosis^25,26^, and is also constantly detected in lungs of patients with idiopathic pulmonary fibrosis^27^. In our results, a relatively high abundance of *Serratia marcescens*, *Staphylococcus aureus*, *Stenotrophomonas maltophilia*, *Acinetobacter baumannii*, *Pseudomonas aeruginosa* and *Candida dubliniensis* were identified in several SARS-CoV-2-positive samples compared with negative samples, which indicated potential coinfection. Nevertheless, these data by themselves could not prove that COVID-19 patients were coinfected by these identified microorganisms; these data have to be carefully interpreted in the clinical context.

### Expression profiles of antibiotic resistance genes

Antimicrobial resistance has become a global issue. Pathogens with antibiotic resistance are increasing and many pathogens are becoming multidrug resistant^28,29^. To characterize antibiotic resistance gene expression profiles, we aligned metatranscriptomic reads against the Comprehensive Antibiotic Resistance Database (CARD)^30^. On average, transcripts of ~124 antibiotic resistance genes were identified in SARS-CoV-2-positive samples, while only ~15 genes were identified in negative samples. According to the CARD, the identified antibiotic resistance genes confer resistance to 28 classes of antibiotics. Almost all resistance gene classes were more abundant in COVID-19 patients compared to healthy individuals. Genes conferring resistance to beta-lactam (including penam, cephalosporin, monobactam, penem, etc.), aminoglycoside, tetracycline, phenicol, rifamycin, fluoroquinolone, and macrolide were the most abundant (Fig. 3d). Overall, the distinct microbiome, emergence of potential coinfection, and the elevated abundance of antibiotic resistance genes provide new data for establishing clinical therapeutic scheme during the treatment for COVID-19 patients.

### Characterization of host response to SARS-CoV-2

**Fig. 4:**
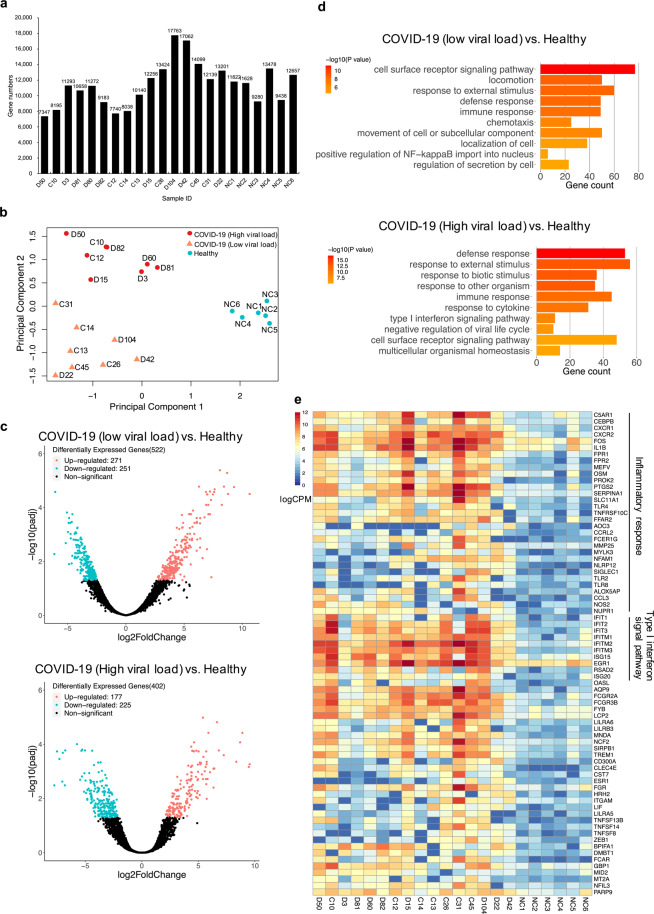
Profiling of host transcriptional response. **a** Bar plot showing gene numbers detected in each sample. **b** MDS plot showing variation among samples based on host transcriptional profiles. **c** Volcano plots showing differentially expressed genes between low SARS-CoV-2 viral load and negative samples (upper), and between high SARS-CoV-2 viral load and negative samples (lower), respectively. Significantly up- and down-regulated genes (padj < 0.05, |log_2_FoldChange| > 1) are highlighted in red and blue, respectively. **d** Bar plots of the most enriched GO terms in low and high SARS-CoV-2 viral load samples, respectively. **e** Heatmap presenting the significantly up-regulated immune response-related genes in SARS-CoV-2-positive samples compared to negative samples.

Distinguishing infection from colonization remains challenging. Because host transcriptional profiling has emerged as a promising diagnostic tool for infectious diseases^31,32^, we next tested whether the host response to SARS-CoV-2 could be simultaneously characterized by TRACE-seq-mediated metatranscriptomic analysis from throat swabs. As shown in Fig. 3a, a substantial percentage of the reads are derived from human, and an average of 11,460 human genes with FPKM > 1 were detected per sample (Fig. 4a; Supplementary Fig. S2a, b). Based on the host gene expression profiles, the relationships between samples were inspected using a multidimensional scaling (MDS) plot (Fig. 4b). As expected, SARS-CoV-2-positive samples were clearly separated from negative samples. In addition, SARS-CoV-2-positive samples could be divided into two subgroups. Further investigation revealed that the two subgroups could also be separated according to viral load (defined by the Ct value of SARS-CoV-2 *ORF1b* region target): subgroup 1 with higher viral load (Ct: 21.97–30.25, except sample D15 (Ct = 32.5)), and subgroup 2 with lower viral load (Ct: 30.51–37.31). To characterize the host responses to different SARS-CoV-2 viral loads, we performed differential gene expression analysis between low SARS-CoV-2 viral load and negative samples, as well as between high viral load and negative samples. We identified 522 differentially expressed genes between low viral load and negative samples, among which 251 genes were up-regulated in low viral load samples (upper panel, Fig. 4c; Supplementary Fig. S2c). We also identified 402 differentially expressed genes between high viral load and negative samples, among which 225 genes were up-regulated in high viral load samples (lower panel, Fig. 4c; Supplementary Fig. S2d). Gene Ontology (GO) enrichment analysis identified that the top up-regulated biological processes in low viral load samples are cell surface receptor signaling pathway, locomotion, response to external stimulus, defense response and immune response, chemotaxis, movement of cell or subcellular component, localization of cell, positive regulation of NF-kappaB import into nucleus, etc., which indicated that host responses in these patients mainly consist of immune response and recruitment of the immune cells. Nevertheless, the top up-regulated biological processes identified in high viral load samples were defense response, response to external stimulus, biotic stimulus and other organism, immune response, and response to cytokine, etc., which indicated that host responses in these patients were dominated by strong immune response (Fig. 4d; Supplementary Fig. S2d). Further investigation of immune response revealed a subset of up-regulated genes in both low and high viral load samples involved in IL1B-associated inflammatory response (*IL1B*, *CXCR1*, *CXCR2*, *FOS*, *C5AR1*, *TLR4*, *CEBPB*, *MEFV*, *FPR1*, *FPR2*, *SLC11A1*, *PROK2*, *PTGS2*, *OSM*, *SERPINA1*, *TNFRSF10C*). Besides, several inflammatory response-related genes (*CCRL2*, *NFAM1*, *FFAR2*, *AOC3*, *MMP25*, *FCER1G*, *SIGLEC1*, *TLR2*, *TLR8*, *MYLK3*) were mainly up-regulated in low viral load samples, and most of them encode proteins functioning as receptors. In addition, several inflammatory response-associated genes (*CCL3*, *NOS2*, *NUPR1*, *ALOX5AP*) were mainly up-regulated in high viral load samples. Moreover, another subset of genes up-regulated in both low and high viral load samples (*ISG15*, *EGR1*, *IFIT1*, *IFIT2*, *IFIT3*, *IFITM1*, *IFITM2*, *IFITM3*, *RSAD2*) were enriched in type I interferon signaling pathway, with two genes (*ISG20*, *OASL*) mainly up-regulated in high viral load samples (Fig. 4e). These results were highly consistent with the previously reported host response to SARS-CoV-2^33–35^. Overall, metatranscriptomic data obtained via TRACE-seq of throat swab samples demonstrate reliable characterization of host transcriptional response to the infection of SARS-CoV-2.

## Discussion

Although next generation sequencing holds a great potential to directly detect known and unknown pathogens including viruses, bacteria, fungi, and parasites in a single application, the laborious and time-consuming steps in traditional RNA library construction procedure hinders its clinical application. As a rapid and convenient one-tube RNA-seq library construction method, TRACE-seq showed comparable performance as traditional RNA library preparation methods in terms of microbiome and host transcriptome profiling (Supplementary Fig. S3), but significantly lower the barrier for extensive application of unbiased RNA-seq in clinical diagnosis. In addition, multiplexing libraries by utilizing Tn5 transposase containing barcoded adaptors could enable sample investigation in a high-throughput manner, particularly when comprehensive surveillance for emerging pathogens is needed during a sudden disease outbreak.

It is very challenging to discriminate pathogens from background commensal microbiota, since substantive bacteria or fungi can colonize multiple body sites of healthy individuals. The microbe present at a relatively higher abundance in patients compared to healthy individuals is often considered as a pathogen, yet the abundance threshold indicating infection is difficult to define based solely on microbiome information. On the other hand, host transcriptional profiling has been reported to distinguish infectious and noninfectious diseases^32^ and to further discriminate between virial and bacterial infections^31^. A previous study integrates host response and unbiased microbe detection for lower respiratory tract infection diagnosis in critically ill adults, using both RNA-seq and DNA-seq but yet lacking antibiotic resistance analysis^3^. Another study characterized microbial gene expression profiles (including antibiotic resistance genes) using nasal and throat swab samples, and host response using blood samples during influenza infection^36^. To our knowledge, this is the first study integrating unbiased pathogen detection, antibiotic resistance, and host response analyses in a single approach with throat swabs from COVID-19 patients. In our results, SARS-CoV-2-positive and -negative samples differed significantly in both microbiome composition and host response. Moreover, TRACE-seq hold the potential to construct a network of microbiome composition, antibiotic resistance, and host response for characterizing the complex host–microbiome interactions. Ideally, TRACE-seq data can be utilized to develop a model combining pathogen diversity metric, antibiotic resistance, and host transcriptional classifier for infectious disease diagnosis. We believe that the integrated information acquired from a TRACE-seq library will deepen our understanding of pathogenesis, improve diagnostic accuracy and more precisely inform optimal antimicrobial treatment for infectious diseases caused by not only SARS-CoV-2 but also other pathogens, and eventually facilitate the utility of metatranscriptomic profiling as a routine diagnostic method.

## Materials and methods

### Ethics statement

The study and use of all samples were approved by the Ethics Committee of Wuhan Institute of Virology (No. WIVH17202001).

### Sample collection and nucleotide extraction

Respiratory specimens (swabs) collected from patients admitted to various Wuhan health care facilities were immediately placed into sterile tubes containing 3 mL of viral transport media (VTM). The swabs were deactivated by heating at 56 °C for 30 min in a biosafety level 2 (BSL 2) laboratory at the Wuhan Institute of Virology in Zhengdian Park with personal protection equipment for biosafety level 3 (BSL 3) laboratory. Total nucleic acids were extracted using QIAamp 96 virus Qiacube HT kit on QIAxtractor Automated extraction (Qiagen, US) following the manufacturer’s instructions.

### TRACE-seq library preparation and sequencing

TRACE-seq libraries were constructed using TruePrep^®^ RNA Library Prep Kit for Illumina (Vazyme, TR502-01) according to the manufacturer’s instructions with several modifications. Firstly, 1/10 volume of total nucleic acids extracted from each swab was used for each library without rRNA removal. Secondly, both random hexamers and oligo(dT)_20_VN primers were used during the reverse transcription process. Thirdly, we used N5 and N7 PCR primer with a final concentration of 0.2 μM during the PCR process. Lastly, after 18 PCR cycles, the library was purified using 0.8× Agencourt AMPure XP beads (Beckman Coulter) and eluted in 20 μL nuclease-free water. The concentration of resulting libraries was determined by Qubit 3.0 fluorometer with the Qubit dsDNA HS Assay kit (Invitrogen) and the size distribution of libraries was assessed by Agilent 2100 Bioanalyzer. Finally, libraries were sequenced on the Illumina Hiseq X10 platform which generated 2 × 150 bp of paired-end raw reads.

### NEBNext Ultra II RNA library preparation

NEBNext Ultra II RNA libraries were constructed using NEBNext Ultra II RNA Library Prep Kit for Illumina (NEB, #E7770S) according to the manufacturer’s instructions.

### Data preprocessing

Raw reads from sequencing were firstly subjected to Trim Galore (v0.6.4_dev) (http://www.bioinformatics.babraham.ac.uk/projects/trim_galore/) for quality control and adaptor trimming. The minimal threshold of quality was 20, and the minimal length of reads to remain was set as 20 nt.

### Host transcriptional profiling analysis

Clean reads were firstly mapped to human rRNA sequences using Bowtie2 (v2.2.9)^37^, and then unmapped reads were mapped to human genome (hg19) and transcriptome using STAR (v2.7.1a)^38^. The FPKM value for annotated genes was calculated by cuffnorm (v2.2.1)^39^, and genes with FPKM > 1 were considered to be expressed. Multidimensional scaling and differential gene expression analysis were conducted using EdgeR (v3.28.1)^40^ with gene count data generated by HTSeq (v0.11.2)^41^. GO enrichment analysis for biological processes was performed by DAVID (v6.8)^42^ with all significantly up-regulated genes as input. Due to the redundancy of enriched GO terms, GO terms and their *P* values were further summarized using REViGO^43^. The top 10 enriched representative GO terms were plotted.

### Discrimination and de novo assembly of SARS-CoV-2

After removal of human reads, the remaining data were aligned to the reference genome of Wuhan-Hu-1 (GenBank accession number: NC_045512) using Bowtie2 (v2.2.9)^37^ for SARS-CoV-2 identification. The coverage and sequencing depth of SARS-CoV-2 genome were calculated by Samtools (v1.9)^44^. On the other hand, to verify that the method could screen for aetiologic agents and obtain pathogen genome, all non-human reads were processed for de novo assembly using MEGAHIT (v1.2.9) with default parameters^45^, and then all contigs were searched against NCBI nt database using blastn for classification^46^. As for accuracy of assembly sequences, contigs determined to come from SARS-CoV-2 were performed blastn (with the parameter “-outfmt 3”) to display the differences with corresponding genome.

### Microbiome analysis

After removing human reads, the remaining reads were subjected to microbial taxonomic classification using Kraken2 (v2.0.8-beta)^47^ with a custom database. To build the custom database, standard RefSeq complete bacterial genomes were downloaded through “kraken2-build --download-library bacteria” and complete genomes of human viruses and genome assemblies of fungi were downloaded from NCBI’s RefSeq and added to the custom database’s genomic library using the “--add-to-library” switch. PCoA of relative abundances of microbial taxa at the genus level was done using cmdscale command in R. Distances between samples were calculated using Morisita-horn dissimilarity index by vegdist command from vegan package version 2.5–6 (https://CRAN.R-project.org/package=vegan). The antibiotic resistance genes were annotated by aligning the filtered metatranscriptomic reads to the CARD. Antibiotic resistance genes with more than 10 completely matching reads were considered. The relative expression of antibiotic resistance genes was determined as RPM (reads per million non-host reads). All corresponding graphs were plotted using R scripts by RStudio (v1.2.5033) (https://rstudio.com/).

### Correlation analysis

The pearson correlation coefficients between TRACE-seq and NEBNext Ultra II RNA kit sequencing data were calculated using function rcorr() in Hmisc package in R, based on microbial relative abundance at genus level called by Kraken2 (v2.0.8-beta)^47^, and host gene expression profiles generated by cuffnorm (v2.2.1)^39^.

## Supplementary information

Supplementary information

## Data Availability

The raw sequence data reported in this paper have been deposited in the Genome Sequence Archive^48^ in National Genomics Data Center^49^, China National Center for Bioinformation/Beijing Institute of Genomics, Chinese Academy of Sciences, under accession number HRA000650 that are publicly accessible at http://bigd.big.ac.cn/gsa-human.
