# Author Correction: Integrated characterization of SARS-CoV-2 genome, microbiome, antibiotic resistance and host response from single throat swabs

**DOI:** 10.1038/s41421-022-00448-5

**Published:** 2022-08-10

**Authors:** Bo Lu, Yi Yan, Liting Dong, Lingling Han, Yawei Liu, Junping Yu, Jianjun Chen, Danyang Yi, Meiling Zhang, Xin Deng, Chao Wang, Runkun Wang, Depeng Wang, Hongping Wei, Di Liu, Chengqi Yi

**Affiliations:** 1 State Key Laboratory of Protein and Plant Gene Research, School of Life Sciences, Peking University, Beijing, 100871 China; 2 Peking-Tsinghua Center for Life Sciences, Peking University, Beijing, 100871 China; 3 CAS Key Laboratory of Special Pathogens, Wuhan Institute of Virology, Center for Biosafety Mega-Science, Chinese Academy of Sciences, Wuhan, Hubei 430071 China; 4 National Virus Resource Center, Wuhan Institute of Virology, Chinese Academy of Sciences, Wuhan, Hubei 430071 China; 5 Computational Virology Group, Center for Bacteria and Viruses Resources and Bioinformation, Wuhan Institute of Virology, Chinese Academy of Sciences, Wuhan, Hubei 430071 China; 6 University of Chinese Academy of Sciences, Beijing, 101409 China; 7 GrandOmics Biosciences, Wuhan, Hubei 430000 China; 8 Department of Gastroenterology, The First Medical Center of PLA General Hospital/Chinese PLA Postgraduate Military Medical School, Beijing, 100853 China; 9 Department of Biomedical Sciences, City University of Hong Kong, Kowloon Tong, Hong Kong, China; 10 First Affiliated Hospital of Xinjiang Medical University, Urumqi, Xinjiang 830054 China; 11 Department of Chemical Biology and Synthetic and Functional Biomolecules Center, College of Chemistry and Molecular Engineering, Peking University, Beijing, 100871 China

Correction to: *Cell Discovery* (2021) 7:19

10.1038/s41421-021-00248-3 published online 30 March 2021

The original version of this article unfortunately contained a mistake. The name of Dengpeng Wang is incorrect. The correct name is Depeng Wang. We apologize for any inconvenience that it may have caused.

