# Supplementary material for: Integrated characterization of SARS-CoV-2 genome, microbiome, antibiotic resistance and host response from single throat swabs
Source: Cell Discov. 2021 Mar 30;7:19. doi: 10.1038/s41421-021-00248-3 (PMC8008776; doi:10.1038/s41421-021-00248-3)
Supplement: Supplementary file 1 — Supplementary information [file 41421_2021_248_MOESM1_ESM.pdf]

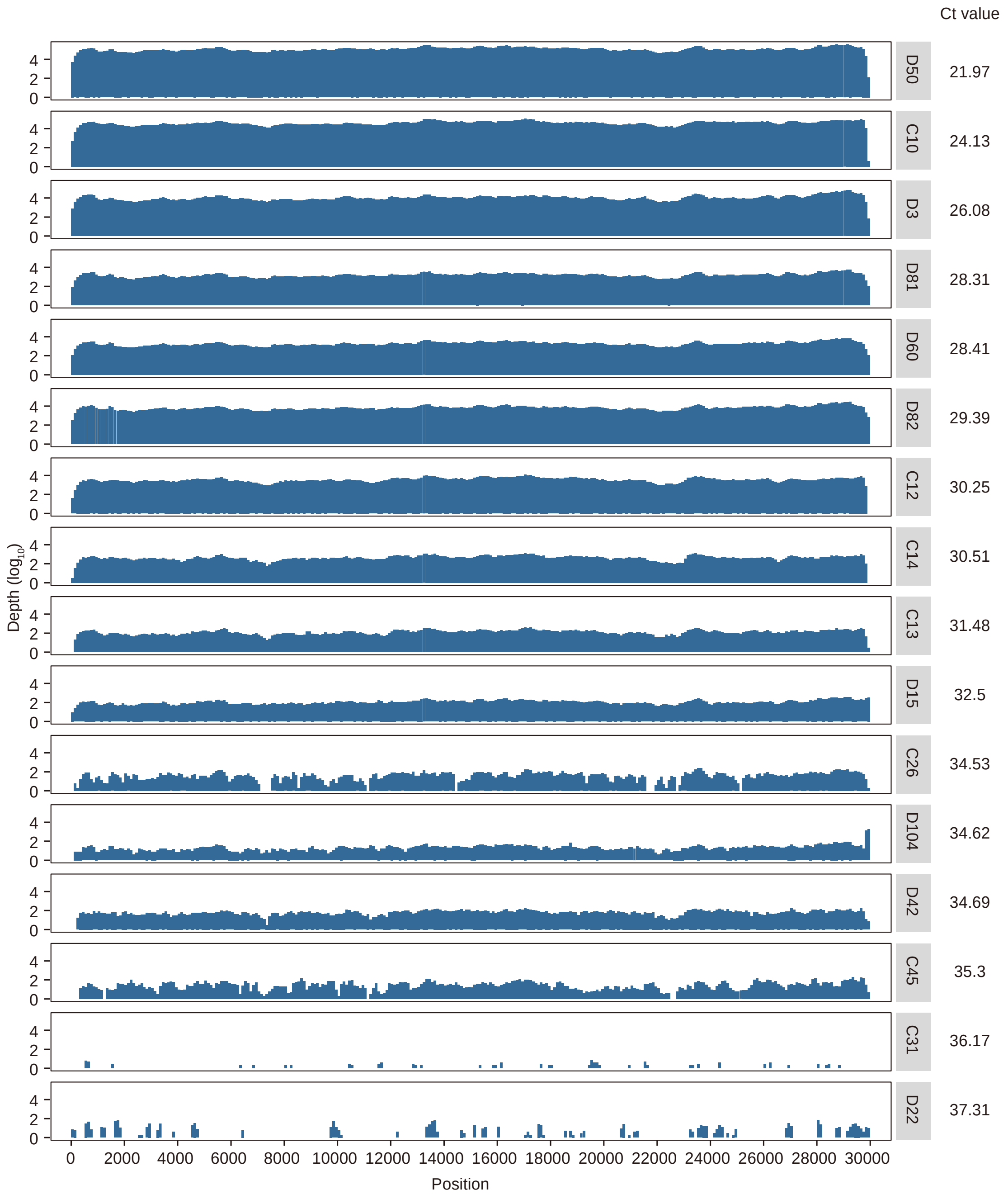

Supplementary Fig. S1 Genome coverage and sequencing depth of SARS-CoV-2 in each sample. The x axis represents the location of SARS-CoV-2 genome, and the y axis represents the log10 depth of every 100 bp of slide window. The samples are arranged from top to bottom according to Ct values, as shown on the right panel.

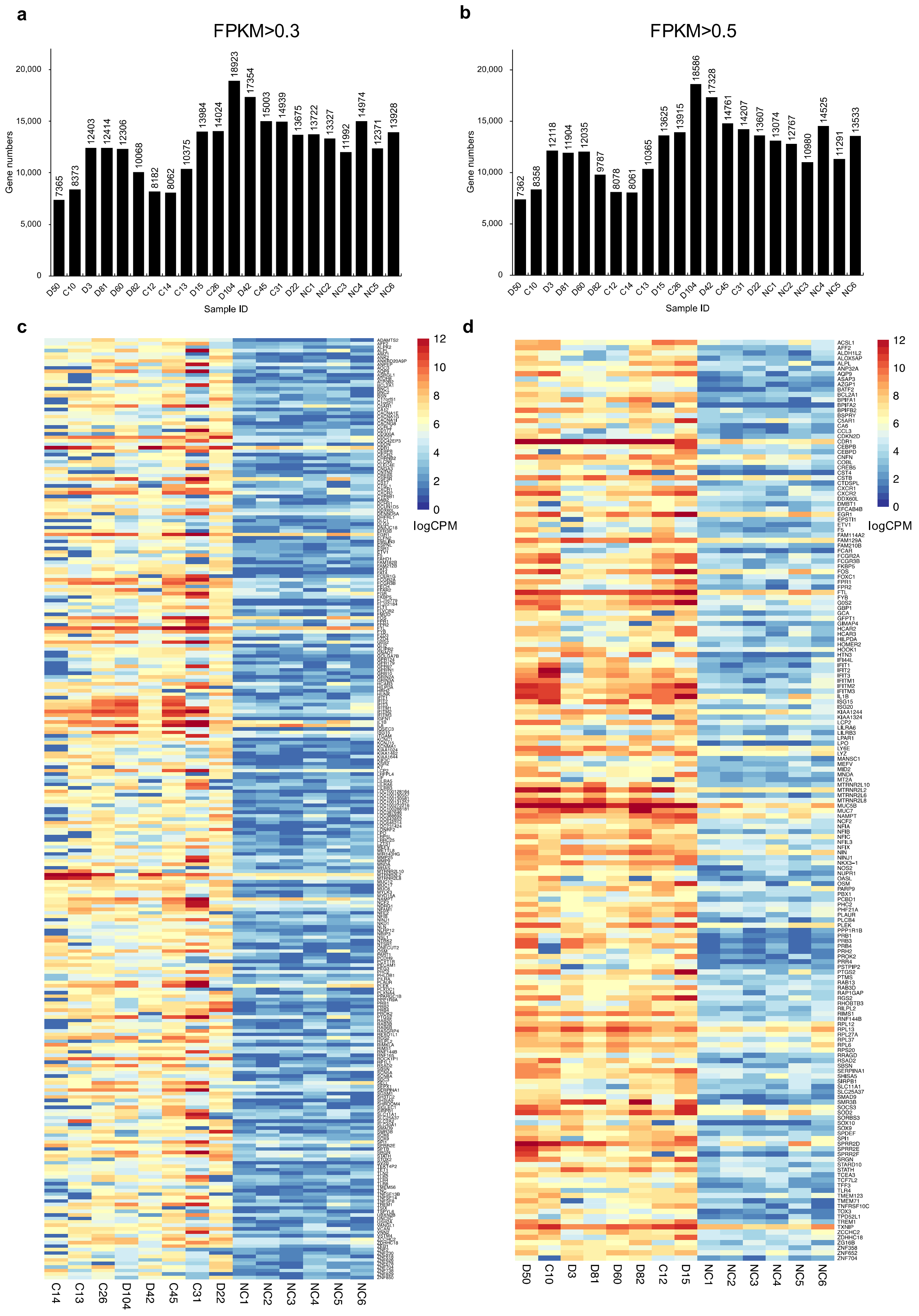

Supplementary Fig. S2 Host gene expression profiling. a Bar plot showing numbers of detected genes with FPKM > 0.3. **b** Bar plot showing numbers of detected genes with FPKM > 0.5. **c** Heatmap showing all genes up-regulated

in low SARS-CoV-2 viral load samples compared to negative samples. **d** Heatmap showing all genes up-regulated in high SARS-CoV-2 viral load samples compared to negative samples.

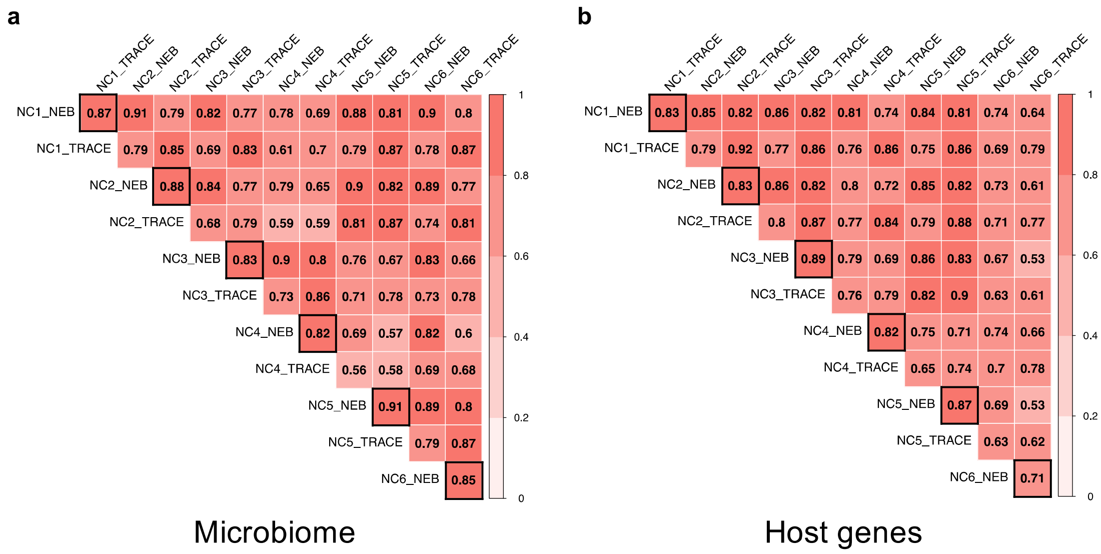

Supplementary Fig. S3 Comparison of the performance of TRACE-seq and NEBNext Ultra II RNA kit on microbiome and host transcriptome profiling. a, b Pearson correlation heatmap of TRACE-seq and NEBNext Ultra II RNA kit sequencing data in terms of microbial community structures (**a**), and host gene expression levels (**b**).

Supplementary Table S1. Mapping results of SARS-CoV-2 for each sample.

Sample	Ct value	Total reads	SARS-CoV-2 reads	Mapping length (bp)	Average depth	Median depth
D50	21.97	42,084,470	33,275,395	29,903	129,900.70	115,569
C10	24.13	27,707,635	9,770,670	29,903	44,065.68	41,608
D3	26.08	45,408,518	3,351,784	29,903	12,810.78	10,407
D81	28.31	56,421,340	464,316	29,903	1,660.15	1,512
D60	28.41	46,154,092	617,330	29,903	2,129.78	1,891
D82	29.39	68,776,916	2,318,220	29,903	7,396.49	6,554
C12	30.25	38,439,824	831,304	29,903	3,983.94	3,530
C14	30.51	16,691,298	101,478	29,862	493.53	447
C13	31.48	31,118,799	32,108	29,827	141.88	131
D15	32.5	43,935,756	35,525	29,896	123.32	106
C26	34.52	38,421,258	11,456	28,232	54.20	46
D104	34.62	85,901,880	10,997	29,814	28.30	21
D42	34.69	24,343,922	17,482	29,679	71.00	66
C45	35.3	43,891,267	8,328	29,549	37.99	27
C31	36.17	31,491,439	150	8,035	0.69	0
D22	37.31	33,458,614	1,252	7,134	4.48	0

Ct: Cycle threshold.
